# Ultrasound-Based Assessment of Shoulder Soft Tissue Alterations in Young Adults Performing Upper Limb Weight Training: A Cross-Sectional Study

**DOI:** 10.3390/jfmk11010023

**Published:** 2026-01-01

**Authors:** Juan José Montoya-Miñano, Carlos Miquel García-de-Pereda-Notario, Luis Palomeque-Del-Cerro, Luis Alfonso Arráez-Aybar

**Affiliations:** 1 Department of Radiology and Rehabilitation, School of Medicine, Complutense University of Madrid, 28040 Madrid, Spain; jjmontoy@ucm.es; 2 Department of Anatomy and Embryology, School of Medicine, Complutense University of Madrid, 28040 Madrid, Spain; arraezla@med.ucm.es; 3 UCM Research Group No. 920202, School of Medicine, Complutense University of Madrid, 28040 Madrid, Spain; 4 Department of Physiotherapy, School of Nursing and Physiotherapy “Salus Infirmorum”, Pontifical University of Salamanca, 28015 Madrid, Spain; lpalomequede@upsa.es; 5 Escuela de Osteopatía de Madrid, 28033 Madrid, Spain

**Keywords:** shoulder injuries, ultrasound diagnosis, acromiohumeral distance, weight training, rotator cuff tendinopathy, long head of the biceps tendon

## Abstract

**Background:** The subacromial space, measured as the acromiohumeral distance (AHD), is a key determinant of shoulder biomechanics and injury risk. Athletes performing repetitive upper-limb resistance training are particularly exposed to cumulative tendon stress. Musculoskeletal ultrasound (US) enables dynamic, cost-effective assessment, yet its role in strength athletes remains underexplored. The aim of this study was to determine whether young adults engaged in regular upper-limb weight training present a narrower acromiohumeral distance and a higher prevalence of ultrasound-detected tendon abnormalities compared with non-weight-training individuals. **Methods:** We conducted a post hoc subanalysis of a cross-sectional cohort of 66 young adults (18–45 years; mean 29.6 ± 9.0 years; 27 men/39 women) evaluated with standardized shoulder US. Participants were classified as weight-training (*n* = 15; 36.2 ± 5.7 years; 11 men/4 women) or non-weight-training (*n* = 51; 27.6 ± 8.8 years; 16 men/35 women). AHD was measured in millimeters, and abnormalities of the supraspinatus, subscapularis, long head of the biceps tendon (LHBT), and subacromial–subdeltoid bursa were recorded. Between-group comparisons used Welch’s t-test or χ^2^/Fisher’s exact test; effect sizes were expressed as Cohen’s d or odds ratios (OR). Multiple testing was corrected with the false discovery rate (FDR). **Results:** Weight-training participants exhibited a significantly smaller AHD (7.13 ± 0.54 vs. 7.49 ± 0.68 mm; t (28) = −2.12, *p* = 0.038; mean difference −0.36 mm, 95% CI −0.70 to −0.03; Cohen’s d = −0.56). Supraspinatus tendinopathy was more prevalent in weight-training athletes (93.3% vs. 41.2%; OR 17.7, 95% CI 2.16–145.8; FDR-adjusted *p* = 0.003). Subscapularis tendinitis (40.0% vs. 17.6%; OR 3.58, 95% CI 1.00–12.88; FDR *p* = 0.14) and LHBT tenosynovitis (20.0% vs. 3.9%; OR 6.82, 95% CI 1.02–45.8; FDR *p* = 0.09) showed non-significant trends. **Conclusions:** Upper-limb weight training in young adults is associated with reduced AHD and a markedly higher prevalence of supraspinatus tendinopathy. Ultrasound proved valuable for early detection of structural and morphological alterations in shoulder soft tissues. Preventive strategies focusing on load management, exercise technique, and targeted strengthening should be prioritized.

## 1. Introduction

Shoulder pain is one of the most frequent musculoskeletal complaints worldwide, with lifetime prevalence up to 66.7% and point prevalence between 7% and 26% in the general population [1]. Within resistance training (RT) sports, the shoulder bears substantial repetitive loading and accounts for as many as 36% of all RT-related injuries [2]. Across systematic strength-training modalities (e.g., weightlifting, powerlifting, and gym-based programs), upper-limb injury prevalence ranges from 12% to 46%, varying by training intensity, modality, and population characteristics [3]. This epidemiological burden highlights the need to better characterize early shoulder alterations in RT practitioners through imaging-based assessment.

Although the shoulder is one of the most vulnerable regions in resistance training (RT) practitioners, there is limited evidence regarding early structural alterations detectable by imaging (e.g., ultrasound) in asymptomatic individuals [4]. In this context, ultrasound-based evaluation of parameters such as the acromiohumeral distance (AHD) and tendon abnormalities of the rotator cuff or the long head of the biceps tendon (LHBT) becomes essential for identifying subclinical structural and morphological alterations in shoulder soft tissues before the onset of symptoms [4]. Upper-limb resistance exercises, including bench press, shoulder press, pull-ups, and curls, impose significant mechanical stress on the glenohumeral joint. Repetitive loading in these movements increases the likelihood of acute and chronic shoulder injuries, particularly affecting tendinous structures of the rotator cuff and the LHBT [5,6]. The unique biomechanics of the shoulder, favoring mobility at the expense of intrinsic stability, make it especially susceptible to load-related syndromes and degenerative changes. The subacromial space, measured as the AHD, plays a central role in these processes, as this anatomical corridor accommodates the supraspinatus tendon, the superior portion of the subscapularis tendon, the subacromial–subdeltoid bursa, and the intra-articular portion of the LHBT before entering the bicipital groove [7,8].

Even minimal narrowing of the acromiohumeral distance (AHD) can increase compression and shear forces, predisposing athletes to impingement syndromes, rotator cuff tendinopathy, and degenerative conditions [9]. The long head of the biceps tendon (LHBT) is considered a versatile but vulnerable structure in shoulder biomechanics, frequently implicated in tendinopathy and instability syndromes, and particularly susceptible to repetitive shear and compressive forces during weight training [10,11]. Musculoskeletal ultrasound (US) provides a dynamic, non-invasive, and cost-effective method to assess the AHD and associated tendon pathology. US has demonstrated high diagnostic accuracy and reliability for evaluating shoulder structures, enabling early identification of both symptomatic and asymptomatic lesions [12,13,14]. The ability of US to detect subclinical changes is clinically valuable, as it may inform preventive strategies for athletes exposed to repetitive loading.

Building on a previously published cohort of young adults assessed with ultrasound [15], the present study specifically analyzes those individuals engaged in upper-limb weight training. Given the demanding mechanical loads and repetitive overhead movements involved in resistance training, it was hypothesized that these athletes may present narrower subacromial spaces and a higher prevalence of tendon pathology compared with non-weight-training participants [16]. The objective of this exploratory post hoc cross-sectional subanalysis was to determine whether young adults engaged in regular upper-limb weight training (Population/Intervention) exhibit a narrower acromiohumeral distance and a higher prevalence of ultrasound-detected tendon abnormalities (supraspinatus, subscapularis, LHBT, and subacromial–subdeltoid bursa) (Outcomes) compared with young adults not performing such training (Comparison).

## 2. Materials and Methods

### 2.1. Study Design

This investigation is an exploratory post hoc subanalysis derived from a previously published observational cross-sectional study of 66 young adults (18–45 years) evaluated with musculoskeletal ultrasound (US) for shoulder soft tissue injuries [15].

This post hoc subanalysis was not pre-specified in the original study protocol and was conceived after completion of the primary data collection, motivated by the clinical relevance of exploring structural shoulder adaptations in participants engaged in upper-limb weight training. Accordingly, all analyses should be considered exploratory.

The present analysis focuses specifically on the subgroup of participants who reported regular upper limb weight training compared with non-weight-training individuals.

### 2.2. Ethics

The original study was conducted in accordance with the Declaration of Helsinki and approved by the Hospital Clínico San Carlos Ethics Committee, Madrid, Spain (reference: 22/148-E_Tesis, approval date: 17 March 2022). All participants provided written informed consent before enrollment [17].

### 2.3. Participants

The original cohort included 66 participants recruited between April and December 2022 through healthcare professionals at Viamed Santa Elena Hospital and faculty members from the Universidad Complutense de Madrid. Inclusion criteria were (1) age between 18 and 45 years, (2) residents of Spain, and (3) either asymptomatic or presenting shoulder pain/structural alteration detectable by ultrasound (e.g., tendinopathy, bursitis, partial tear). Exclusion criteria included tumors, infections, recent upper limb or cervical spine surgery, vascular disease, congenital malformations, or fever at the time of evaluation [18].

For this study, each participant underwent ultrasound evaluation of a single shoulder, selected according to clinical relevance (symptomatic side when pain was present, or dominant side in asymptomatic individuals).

For the purposes of this subanalysis, participants were divided into two groups: as weight training (*n* = 15) or non-weight-training (*n* = 51). (1) Weight-training group, defined as individuals performing ≥ 2 structured upper-limb resistance-training sessions per week for at least 6 consecutive months, including multi-joint and single-joint exercises (e.g., bench press, overhead press, pull-ups, curls).

In addition, a six-week monitoring period was conducted to document each participant’s upper-limb training characteristics, ensuring that at least eight sessions were completed during this timeframe.

Participants were further classified into two subtypes according to their predominant training goals:

- Group A—Strength/Hypertrophy Training: focused on maximal strength or muscle hypertrophy (typically 4–6 sets of 6–10 repetitions, high load, moderate speed, 2–3 sessions per week).
- Group B—Functional/General Conditioning: focused on mixed or functional conditioning routines (typically 2–4 sets of 10–15 repetitions, moderate load, variable movements, 2 sessions per week).

This threshold aligns with international physical activity recommendations that advocate at least two sessions of muscle-strengthening activities per week for adults [19]. (2) Non-weight-training group, including participants not engaged in regular upper-limb resistance training.

The exact long-term weekly training frequency beyond the predefined threshold was not retrospectively quantified, which is acknowledged as a study limitation. However, participants in the weight-training group were prospectively monitored over a six-week period and reported their average number of weekly training sessions. These data were used to provide a descriptive characterization of recent training exposure and are summarized in Table 1; however, training frequency was not treated as a primary analytical variable.

Participants performing CrossFit or similar functional resistance programs were also included in the weight-training group, provided that their routines involved at least two structured upper-limb resistance sessions per week for a minimum of six months.

Given the modest size of the weight-training subgroup, all statistical comparisons should be considered exploratory and interpreted with caution, as only effects of large magnitude are likely to be detectable.

Table 1 summarizes the training and physical activity characteristics of both groups during the six-week monitoring period.

This table provides an overview of the main exposure parameters recorded during the six-week monitoring period, including training modality, frequency, volume, and adherence in the weight-training group, as well as the typical low-intensity activities reported by non-weight-training participants. Detailed individual data are provided in Supplementary Tables S1 and S2.

For the non-weight-training group, “weekly sessions” refers to general physical activity sessions (e.g., walking, recreational activities, or aerobic exercise) and not to structured resistance training.

### 2.4. Participant Flow

**Figure 1:**
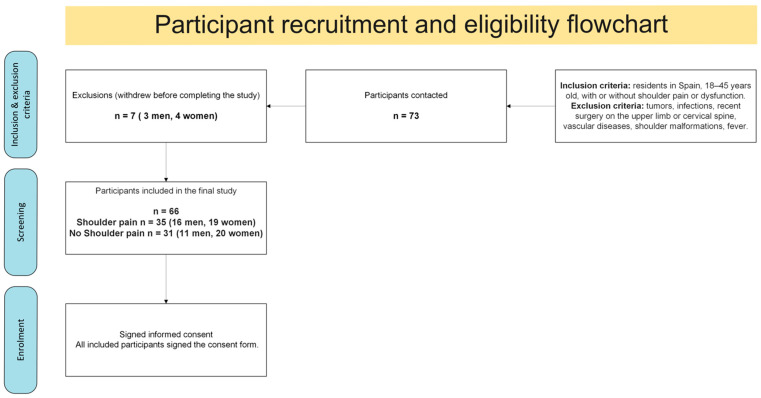
Flow diagram of participant recruitment, eligibility assessment, exclusions, and final enrollment.

Figure 1 shows the recruitment process, eligibility assessment, exclusions, and final enrollment of participants from the original cohort (*n* = 66). The present subanalysis focused on those engaged in upper-limb weight training (*n* = 15) compared with non-weight-training individuals (*n* = 51).

### 2.5. Ultrasound Procedure

All participants underwent a high-resolution musculoskeletal shoulder US (Canon CUS-AA000 Aplio a; Canon Medical Systems Corporation, Ōtawara, Japan).

Ultrasound examinations were performed using a high-frequency linear-array transducer, as per standard musculoskeletal shoulder imaging protocols.

All examinations were performed using the manufacturer’s standardized musculoskeletal shoulder preset, with imaging parameters optimized for superficial soft-tissue assessment.

All ultrasound assessments were performed by a single radiologist with nine years of clinical experience, who was blinded to participants’ group allocation to prevent bias. Measurements were obtained with participants seated, trunk in a neutral posture, and arm resting alongside the body in an anatomical position. The transducer was positioned on the anterolateral aspect of the shoulder, and the shortest linear distance between the inferior border of the acromion and the superior aspect of the humeral head was recorded automatically in millimeters by the ultrasound system.

The acromiohumeral distance (AHD) was measured with participants seated, arm resting alongside the body, and the transducer placed on the anterolateral shoulder. The shortest linear distance from the inferior acromial border to the superior aspect of the humeral head was recorded in millimeters.

Each AHD measurement was repeated three times in a subsample of 15 participants to assess intra-observer reliability. The intraclass correlation coefficient (ICC) was 0.95 (95% CI: 0.91–0.98), confirming excellent reproducibility. Based on these data, the standard error of measurement (SEM) was 0.16 mm, and the minimum detectable change (MDC, 95% confidence) was 0.44 mm.

In addition, ultrasound evaluation of shoulder structures included the supraspinatus tendon, subscapularis tendon, long head of the biceps tendon (LHBT), and the subacromial–subdeltoid bursa. Pathological findings were recorded according to established diagnostic criteria [20].

Ultrasound-detected pathologies were defined according to established musculoskeletal ultrasound consensus criteria. Supraspinatus tendinopathy was defined as tendon thickening and/or hypoechogenicity with loss of the normal fibrillar pattern, with or without focal intrasubstance abnormalities. Long head of the biceps tendon (LHBT) tenosynovitis was defined as the presence of anechoic or hypoechoic fluid surrounding the tendon within the bicipital groove, with or without synovial sheath thickening. Subacromial–subdeltoid bursitis was defined as bursal fluid distension and/or synovial thickening exceeding normal physiological limits. Partial-thickness rotator cuff tears were identified by focal discontinuity or hypoechoic defects within the tendon substance, not extending through the full tendon thickness.

No formal grading or severity classification of supraspinatus tendinopathy was applied; tendon abnormalities were recorded as present or absent based on structural ultrasound criteria.

### 2.6. Statistical Analysis

Continuous variables were expressed as mean ± standard deviation (SD), and categorical variables as frequencies and percentages. Normality was assessed using the Shapiro–Wilk test (*p* > 0.05 for AHD in both groups), which justified the use of parametric analyses.

Primary analysis. Between-group comparisons of acromiohumeral distance (AHD) were performed using an independent samples *t*-test with Welch’s correction for unequal variances, reporting the Welch-adjusted degrees of freedom. Only the Welch-adjusted statistics are presented.

Adjusted models. Analysis of covariance (ANCOVA) was conducted with group status (weight training vs. non-weight-training) as the main factor and age, sex, and shoulder pain as covariates.

Although no participant reported clinically relevant shoulder pain at baseline, pain-related data were collected using the Short-Form McGill Pain Questionnaire and treated as a continuous variable. This approach allowed for adjustment for subclinical pain-related variability in the statistical models.

These variables were selected because of their established influence on AHD and tendon pathology. Other potential confounders, such as overall physical activity level or limb dominance, were not included, which represents a limitation to be addressed in future studies. Shoulder pain was included as a covariate not as a causal factor but to control for its potential modulatory influence on posture, muscle activation, and AHD measurements, thereby reducing variability unrelated to structural alterations.

Association with lesions. Multivariable logistic regression models were fitted (e.g., LHBT tenosynovitis ~ AHD + group status + age + sex + pain), reporting odds ratios (OR) with 95% confidence intervals.

Effect sizes. Effect sizes were systematically calculated to complement significance testing. Cohen’s *d* was used to quantify the standardized mean difference in AHD, interpreted as small (0.2), medium (0.5), or large (0.8) [21].

For categorical outcomes, ORs with 95% CI were reported as measures of association and interpreted following established benchmarks (OR ≈ 1.5 small, ≈2 medium, ≥3 large) [22].

Multiple testing. To control for the risk of type I error, the Benjamini–Hochberg false discovery rate (FDR) procedure was applied across all analyses.

Exploratory approach. Given the modest size of the weight-training subgroup, all inferential analyses were considered exploratory, and results were interpreted with caution. In this context, only effects of large magnitude are likely to be detectable.

Data management and software. Ultrasound and questionnaire data were entered into a standardized database (Microsoft Excel v16.0, Microsoft Corp., USA), independently verified by two investigators, and exported for analysis. All statistical analyses were conducted using R v4.3.1 (packages: stats, car, multcomp). A two-tailed *p* < 0.05 was considered statistically significant, unless otherwise adjusted by FDR [23,24].

## 3. Results

### Weight Training vs. Non-Weight Training

A total of 66 participants were analyzed (27 males, 39 females; mean age 29.6 ± 9.0 years). Among them, 15 participants (22.7%) reported regular upper-limb weight training, while the remaining 51 (77.3%) were classified as non-weight-training. Table 2 summarizes the sociodemographic characteristics of the participants stratified by training status.

The mean acromiohumeral distance (AHD) in the total sample was 7.4 ± 0.7 mm, consistent with reference values previously reported in both healthy and symptomatic populations [25].

When stratified by group, weight-training participants showed a significantly smaller AHD compared with non-weight-training participants (7.13 ± 0.54 mm vs. 7.49 ± 0.68 mm; Welch’s t-test: mean difference −0.37 mm, 95% CI −0.70 to −0.03; *p* = 0.038; Cohen’s d = −0.56). This represents a moderate effect size. Importantly, this association remained significant after adjustment for age, sex, and pain in the ANCOVA model. Age acted as a modulatory covariate—given that weight-training participants were on average older—yet this did not fully account for the between-group differences. The reduction in subacromial space is clinically relevant, as narrowing of the AHD has been associated with impingement syndromes and rotator cuff tendinopathy in overhead or strength athletes [9].

Regarding ultrasound-detected lesions, supraspinatus tendinopathy was markedly more frequent in the weight-training group than in the non-weight-training group (93.3% vs. 41.2%; OR 17.7, 95% CI 2.16–145.8; unadjusted *p* = 0.0007; FDR-adjusted *p* = 0.003). This corresponds to a large effect size, suggesting a strong association between weight training and structural alterations in the supraspinatus tendon. Subscapularis tendinitis was observed in 40.0% of weight-training participants versus 17.6% of non-weight-training participants (OR 3.58, 95% CI 1.00–12.88; unadjusted *p* = 0.096; FDR-adjusted *p* = 0.14), indicating a medium effect size although not statistically significant after correction. LHBT tenosynovitis was detected in 20.0% of weight-training participants compared with 3.9% of non-weight-training participants (OR 6.82, 95% CI 1.02–45.8; unadjusted *p* = 0.060; FDR-adjusted *p* = 0.09), a finding compatible with a large effect size but remaining non-significant after correction. Subacromial–subdeltoid bursitis was observed in one weight-training participant (6.7%) but in none of the non-weight-training individuals (*p* = 0.21).

A detailed comparison of AHD and ultrasound-detected lesions between groups is provided in Table 3.

**Figure 2:**
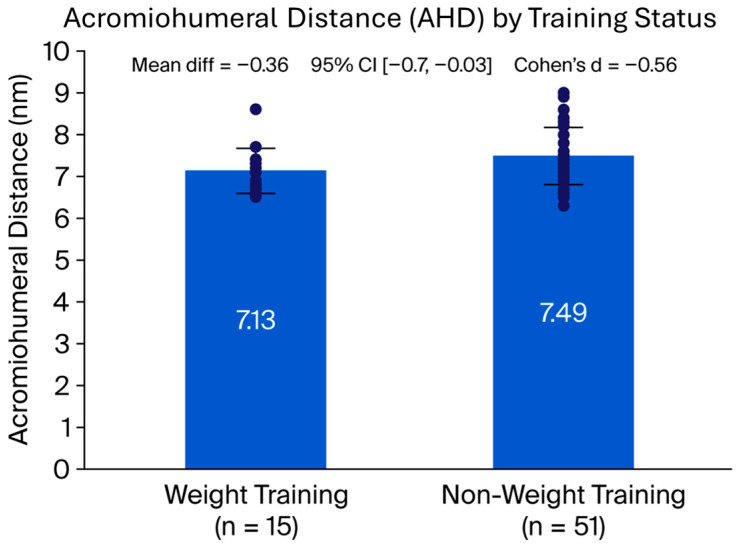
Comparison of acromiohumeral distance (AHD) between groups. Bars represent mean values (± SD) in weight-training (*n* = 15) and non-weight-training (*n* = 51) participants, assessed by ultrasound. Error bars indicate standard deviations. Abbreviations: AHD, acromiohumeral distance; SD, standard deviation.

These between-group differences are illustrated in Figure 2.

These findings indicate that regular upper-limb weight training is associated with a significant narrowing of the acromiohumeral space and with a markedly higher prevalence of supraspinatus tendinopathy, whereas the observed trends for subscapularis and LHBT involvement did not reach statistical significance after FDR adjustment. Within the weight-training subgroup, almost all participants presented supraspinatus tendinopathy (93.3%), and more than half exhibited multi-tendon involvement (subscapularis, LHBT, or bursa), suggesting a clustering of structural soft-tissue alterations in this population.

Multivariable models adjusted for age, sex, and pain confirmed that the reduction in AHD among weight-training participants remained significant, whereas associations with subscapularis and LHBT involvement did not achieve significance after adjustment (Table 4).

## 4. Discussion

This exploratory subanalysis demonstrated that young adults performing regular upper limb weight training presented a significantly narrower acromiohumeral distance (AHD) and a markedly higher prevalence of supraspinatus tendinopathy compared with non-weight-training individuals. These results are consistent with the vulnerability of the supraspinatus tendon to structural and morphological alterations associated with repetitive loading in overhead and resistance exercises, supporting previous literature that identifies this structure as a primary site of degeneration and pain generation [9]. Although that meta-analysis focused mainly on older adults, similar mechanical and anatomical mechanisms may contribute to early tendon alterations in younger strength-training populations.

The association between weight training and supraspinatus pathology is biomechanically plausible. Exercises such as bench press, overhead press, or pull-ups repeatedly place the supraspinatus under combined compressive and tensile stress within the subacromial space. The concomitant reduction in AHD observed in the weight-training group strengthens the concept that narrowing of the subacromial corridor contributes to rotator cuff tendinopathy, as suggested in imaging and cadaveric studies [8].

Although the between-group difference in acromiohumeral distance reached statistical significance, its magnitude (0.36 mm) did not exceed the previously reported minimum detectable change (MDC = 0.44 mm). This finding indicates that the observed difference may not be reliably detectable at the individual level and should therefore not be interpreted as a clinically meaningful change per se. Rather, it reflects a consistent group-level structural difference that may represent early or subclinical adaptations associated with upper-limb weight training. From a measurement perspective, this distinction underscores the importance of differentiating statistical significance from measurement sensitivity and clinical relevance when interpreting small changes in AHD.

Although the weight-training group included a higher proportion of men and was slightly older than the comparison group, these variables were statistically controlled in the adjusted models, and previous evidence indicates that age and sex exert minimal influence on acromiohumeral distance and rotator cuff integrity in adults under 45 years [25].

Regarding the long head of the biceps tendon (LHBT), a higher prevalence of tenosynovitis was detected in weight-training participants (20% vs. 3.9%), although this trend did not reach statistical significance after multiple testing correction. Despite this, the effect size was large, and clinical interpretation remains relevant. The LHBT is known to be susceptible to shear forces and entrapment when the subacromial space narrows, which may explain the observed tendency toward greater involvement in this subgroup [10,11].

The descriptive analysis also revealed that nearly all weight-training participants showed supraspinatus tendinopathy, with more than half presenting multi-structure involvement (subscapularis, LHBT, or bursa). This clustering of lesions highlights the cumulative stress imposed by high-load resistance training and suggests that structural and morphological alterations in these athletes rarely affects a single structure in isolation. Preventive strategies focused on optimizing technique, managing load progression, and strengthening the rotator cuff appear essential to mitigate this risk [2].

The inclusion of a structured six-week monitoring period allowed a more precise characterization of training exposure, differentiating between strength/hypertrophy and functional conditioning programs. This refinement helps to contextualize the observed shoulder adaptations and may partly explain inter-individual variability in tendon findings.

This complementary dataset (Supplementary Table S1) specifically details training type, weekly volume, and adherence.

From a clinical perspective, musculoskeletal ultrasound proved to be a sensitive tool for detecting subtle structural and morphological alterations in shoulder soft tissues, even in asymptomatic individuals. The ability to identify subacromial narrowing and tendon abnormalities before the onset of symptoms is highly valuable, as it offers the opportunity for early intervention, load adjustment, and individualized rehabilitation [12,13].

Incorporating ultrasound into regular screening protocols for strength athletes could help prevent progression toward symptomatic injury.

The high prevalence of ultrasound-detected supraspinatus tendinopathy observed in asymptomatic participants should be interpreted with caution. These findings do not necessarily indicate clinical disease, but rather reflect subclinical or early structural adaptations that may occur in physically active individuals. While musculoskeletal ultrasound allows sensitive detection of subtle tissue changes, isolated imaging findings should not be equated with pathology or used to justify intervention in the absence of symptoms. Therefore, the potential risk of overdiagnosis underscores the importance of integrating ultrasound findings within a comprehensive clinical assessment, emphasizing their role in monitoring tissue adaptation and guiding preventive strategies rather than establishing standalone diagnoses.

Given the well-established benefits of resistance training for musculoskeletal health and overall physical performance, the findings of the present study should not be interpreted as a rationale to discourage participation in such activities. Instead, they highlight the importance of implementing preventive strategies aimed at optimizing load management, exercise technique, and shoulder-specific conditioning. The early detection of subclinical structural alterations through musculoskeletal ultrasound may allow timely interventions, reducing the risk of progression toward symptomatic injury while supporting safe and sustainable engagement in resistance training.

Nevertheless, several limitations should be acknowledged. First, only one shoulder per participant was examined, which limits extrapolation to bilateral shoulder involvement. Second, the weight-training subgroup was modest in size (*n* = 15), which reduces statistical power and generalizability. In this context, only effects of large magnitude are likely to be detectable. In addition, information on training frequency, intensity, and specific exercise modalities was not collected, which may influence both the magnitude and distribution of shoulder alterations. Replication of these findings in larger, sport-specific cohorts with detailed training variables and longitudinal follow-up is necessary to validate and extend the present results [26].

In addition, the odds ratios derived from logistic regression analyses should be interpreted with caution. Several estimates were associated with wide confidence intervals, likely reflecting sparse data and the modest size of the weight-training subgroup. This statistical instability limits the precision of effect size estimation and increases uncertainty around the true magnitude of the associations. Although conventional logistic regression was deemed appropriate for the exploratory aims of this study, future investigations with larger samples could benefit from alternative approaches, such as penalized or exact logistic regression methods, to improve the robustness of risk estimates.

However, the inclusion of a six-week monitored period with verified training exposure mitigates some of the uncertainties previously noted in training characterization.

Nevertheless, short-term exposure was objectively documented through a six-week monitoring protocol (see Supplementary Table S1 for training profiles and adherence).

Although pain-related data were collected using the Short-Form McGill Pain Questionnaire, the present study was not powered to analyze symptom severity or its relationship with acromiohumeral distance. Therefore, pain was not considered a primary outcome, and results should be interpreted as reflecting structural rather than clinical associations.

It should also be noted that pain was not used as a grouping variable in this study. All participants completed the Short-Form McGill Pain Questionnaire [27]; however, previous evidence from our research group [15], demonstrated that shoulder pain is not necessarily correlated with the presence of ultrasound-detected soft-tissue lesions. Consequently, pain was not considered a discriminating factor between groups, as the main objective was to compare structural alterations according to exposure to upper-limb resistance training, regardless of symptom status.

In conclusion, this subanalysis provides evidence that upper limb weight training is associated with narrowing of the acromiohumeral space and increased supraspinatus tendinopathy prevalence, with a non-significant but clinically meaningful trend toward greater LHBT involvement. Musculoskeletal ultrasound emerges as a valuable tool for monitoring shoulder health in strength athletes, reinforcing its role in early detection of structural and morphological alterations, preventive strategies, and individualized rehabilitation programs.

Furthermore, the specific type and objective of resistance training (e.g., hypertrophy, maximal strength, muscular endurance, or functional training such as CrossFit) were not differentiated. This lack of granularity limits interpretation, as different modalities may exert distinct mechanical loads on shoulder structures.

In addition, the exact weekly training frequency (i.e., number of resistance sessions per week) among participants in the weight-training group was not quantified, which may influence the degree of shoulder structural adaptation and should be addressed in future studies.

## 5. Conclusions

This exploratory subanalysis indicates that young adults engaged in regular upper limb weight training exhibit a significantly narrower acromiohumeral distance and a markedly higher prevalence of supraspinatus tendinopathy compared with non-weight-training individuals. A non-significant but clinically relevant trend toward greater involvement of the long head of the bicep’s tendon was also observed.

These findings suggest that repetitive upper-limb resistance exercises may predispose athletes to subacromial narrowing and multi-tendon structural alterations, highlighting the supraspinatus as the structure most consistently affected. Musculoskeletal ultrasound proved to be a reliable and clinically valuable tool for identifying these alterations, even in asymptomatic individuals, supporting its role in early screening and longitudinal monitoring of athletes exposed to intensive upper-limb training.

Preventive strategies, such as optimizing exercise technique, managing load progression, and implementing targeted rotator cuff strengthening, should be prioritized to reduce injury risk in this population. Future studies with larger, sport-specific cohorts and detailed training variables are warranted to confirm these associations and refine preventive and rehabilitative approaches. These results may guide physiotherapists, sports medicine practitioners, and trainers in designing preventive interventions tailored to the specific demands of strength athletes.

## Figures and Tables

**Table 1:** Summary of training and physical activity characteristics of participants.

Variable	Weight Training (*n* = 15)	Non-Weight Training (*n* = 51)	Source
Training modality	Strength/Hypertrophy (*n* = 10); Functional/General Conditioning (*n* = 5)	Walking, Pilates, yoga, cycling, swimming	Tables S1 and S2
Weekly sessions (mean ± SD)	3.7 ± 1.1	2.6 ± 0.8	Tables S1 and S2
Average sets × repetitions	4.8 × 9.5	—	Table S1
Mean load (%1RM)	75–85% (estimated)	—	Table S1
Adherence (%)	93 ± 8	—	Table S1
Main exercises	Bench press, overhead press, pull-ups, squats	Walking, Pilates, yoga, cycling	Tables S1 and S2
Training experience (years, mean ± SD)	10.2 ± 4.5	—	Table S1

Note: Data refer to the six-week monitoring period used to verify training exposure in both groups. Detailed individual data are presented in Supplementary Tables S1 and S2.

**Table 2:** Sociodemographic characteristics of participants by training status.

Variable	Total (*n* = 66)	Weight Training (*n* = 15)	Non-Weight Training (*n* = 51)	Between-Group Comparison
Age, mean ± SD (years)	29.6 ± 9.0	36.2 ± 5.7	27.6 ± 8.8	t (28) = 3.90, *p* < 0.001
Male, *n* (%)	27 (40.9%)	11 (73.3%)	16 (31.4%)	χ^2^ = 8.07, *p* = 0.004
Female, *n* (%)	39 (59.1%)	4 (26.7%)	35 (68.6%)	
Regular physical activity, *n* (%) ^1^	38 (57.6%)	15 (100%)	23 (45.1%)	Fisher’s exact test, *p* = 0.001; φ = 0.42 (large)

^1^ Defined as engaging in ≥2 weekly sessions of moderate-to-vigorous physical activity, in line with [19]. Note: Values are expressed as mean ± standard deviation (SD) or frequency (%). None of the participants reported shoulder pain at baseline.

**Table 3:** Comparison of ultrasound variables between weight-training and non-weight-training participants.

Variable	Weight Training (*n* = 15)	Non-Weight Training (*n* = 51)	Between-Group Comparison	Effect Size
AHD, mean ± SD (mm)	7.13 ± 0.54	7.49 ± 0.68	Welch’s t-test, mean diff. −0.36 mm (95% CI −0.70 to −0.03), *p* = 0.038	|d| = 0.56 (moderate)
Supraspinatus tendinopathy, *n* (%)	14 (93.3%)	21 (41.2%)	χ^2^/Fisher, FDR *p* < 0.001	|OR| = 17.7 (95% CI 2.16–145.8, large)
Subscapularis tendinitis, *n* (%)	6 (40.0%)	9 (17.6%)	FDR *p* = 0.140	|OR| = 3.58 (95% CI 1.00–12.88, medium)
LHBT tenosynovitis, *n* (%)	3 (20.0%)	2 (3.9%)	FDR *p* = 0.090	|OR| = 6.82 (95% CI 1.02–45.8, large)
Subacromial–subdeltoid bursitis, *n* (%)	1 (6.7%)	0 (0%)	*p* = 0.210	—

Note: AHD = acromiohumeral distance; LHBT = long head of the bicep’s tendon. OR = odds ratio; CI = confidence interval; FDR = false discovery rate. Effect sizes are reported in absolute values (|d|, |OR|).

**Table 4:** Adjusted models for AHD and tendon abnormalities.

Outcome Variable	Model (Adj. Age, Sex, Pain)	Adjusted Effect (95% CI)	Test Statistic	Effect Size	FDR-Adjusted *p*
AHD (mm)	ANCOVA	−0.36 mm (95% CI −0.70 to −0.03)	Welch’s t(28) = −2.12	Cohen’s d = −0.56 (moderate)	0.038
Supraspinatus tendinopathy	Logistic regression	OR 17.7 (95% CI 2.16–145.8)	χ^2^ = 11.5	OR 17.7 (large)	0.003
Subscapularis tendinitis	Logistic regression	OR 3.58 (95% CI 1.00–12.88)	χ^2^ = 3.6	OR 3.58 (medium)	0.140
LHBT tenosynovitis	Logistic regression	OR 6.82 (95% CI 1.02–45.8)	χ^2^ = 3.5	OR 6.82 (large)	0.090

Note: All models adjusted for age, sex, and pain. OR = odds ratio; CI = confidence interval; SE = standard error; FDR = false discovery rate; η^2^ = partial eta squared; R^2^ = Nagelkerke pseudo-R^2^. Raw *p* = unadjusted *p*-value; FDR-*p* = *p* adjusted for multiple testing.

## Data Availability

The data presented in this study are available on reasonable request from the corresponding author. The data are not publicly available due to privacy restrictions.

## Supplementary Materials

The following supporting information can be downloaded at: https://www.mdpi.com/article/10.3390/jfmk11010023/s1, Table S1: Training characteristics of weight-training participants; Table S2: Physical activity profile of non-weight-training participants.

## References

[1] Lucas J., van Doorn P., Hegedus E., Lewis J., van der Windt D. (2022). A systematic review of the global prevalence and incidence of shoulder pain. BMC Musculoskelet. Disord..

[2] Kolber M.J., Beekhuizen K.S., Cheng M.S., Hellman M.A. (2010). Shoulder injuries attributed to resistance training: A brief review. J. Strength Cond. Res..

[3] Serafim T.T., de Oliveira E.S., Maffulli N., Migliorini F., Okubo R. (2023). Which resistance training is safest to practice? A systematic review. J. Orthop. Surg. Res..

[4] Tagliafico A., Cadoni A., Bignotti B., Martinoli C. (2014). High-resolution ultrasound of rotator cuff and biceps reflection pulley in non-elite junior tennis players: Anatomical study. BMC Musculoskelet. Disord..

[5] Jacob J., O’Connor P., Pass B. (2022). Muscle Injury Around the Shoulder. Semin. Musculoskelet. Radiol..

[6] Li L., Ren F., Baker J.S. (2021). The Biomechanics of Shoulder Movement with Implications for Shoulder Injury in Table Tennis: A Minireview. Appl. Bionics Biomech..

[7] Bakhsh W., Nicandri G. (2018). Anatomy and Physical Examination of the Shoulder. Sports Med. Arthrosc. Rev..

[8] Kennedy M.S., Nicholson H.D., Woodley S.J. (2017). Clinical anatomy of the subacromial and related shoulder bursae: A review of the literature. Clin. Anat..

[9] Leong H., Fu S., He X., Oh J., Yamamoto N., Yung S. (2019). Risk factors for rotator cuff tendinopathy: A systematic review and meta-analysis. J. Rehabilitation Med..

[10] Diplock B., Hing W., Marks D. (2023). The long head of biceps at the shoulder: A scoping review. BMC Musculoskelet. Disord..

[11] Ahrens P.M., Boileau P. (2007). The long head of biceps and associated tendinopathy. J. Bone Jt. Surgery. Br. Vol..

[12] Sher J., Uribe J., Posada A., Murphy B., Zlatkin M. (1995). Abnormal findings on magnetic resonance images of asymptomatic shoulders. J. Bone Jt. Surg..

[13] Wengert G.J., Schmutzer M., Bickel H., Sora M.-C., Polanec S.H., Weber M., Schueller-Weidekamm C. (2019). Reliability of high-resolution ultrasound and magnetic resonance arthrography of the shoulder in patients with sports-related shoulder injuries. PLoS ONE.

[14] Pan G. (2024). Current status of dynamic musculoskeletal ultrasound for application to treatment of orthopedic diseases. Am. J. Transl. Res..

[15] García-De-Pereda-Notario C.M., Palomeque-Del-Cerro L., García-Mata R., Rodriguez-Isarn M., Rodriguez-Isarn H., Arráez-Aybar L.A. (2025). Ultrasound-Based Anatomical Assessment of the Most Common Shoulder Soft Tissue Injuries in Young Adults. Healthcare.

[16] Noschajew E., Azesberger A., Rittenschober F., Windischbauer A., Gruber M.S., Ortmaier R. (2022). The Effect of Strength Training on Undetected Shoulder Pathology in Asymptomatic Athletes: An MRI Observational Study. Sports.

[17] World Medical Association (2002). World Medical Association Declaration of Helsinki. Ethical principles for medical research involving human subjects. Nurs. Ethic.

[18] Cadogan A., Laslett M., Hing W.A., McNair P.J., Coates M.H. (2011). A prospective study of shoulder pain in primary care: Prevalence of imaged pathology and response to guided diagnostic blocks. BMC Musculoskelet. Disord..

[19] (2020). WHO Guidelines on Physical Activity and Sedentary Behaviour.

[20] Requejo-Salinas N., Lewis J., Michener L.A., La Touche R., Fernández-Matías R., Tercero-Lucas J., Camargo P.R., Bateman M., Struyf F., Roy J.-S. (2022). International physical therapists consensus on clinical descriptors for diagnosing rotator cuff related shoulder pain: A Delphi study. Braz. J. Phys. Ther..

[21] Cohen J. (2009). Statistical Power Analysis for the Behavioral Sciences.

[22] Chen H., Cohen P., Chen S. (2010). How Big is a Big Odds Ratio? Interpreting the Magnitudes of Odds Ratios in Epidemiological Studies. Commun. Stat.—Simul. Comput..

[23] Ward J.H. (1963). Hierarchical Grouping to Optimize an Objective Function. J. Am. Stat. Assoc..

[24] Lakens D. (2013). Calculating and reporting effect sizes to facilitate cumulative science: A practical primer for *t*-tests and ANOVAs. Front. Psychol..

[25] Kim K., Kim H.G., Song D., Yoon J.Y., Chung M.E. (2016). Ultrasound Dimensions of the Rotator Cuff and Other Associated Structures in Korean Healthy Adults. J. Korean Med. Sci..

[26] Hackshaw A. (2008). Small studies: Strengths and limitations. Eur. Respir. J..

[27] Melzack R. (1987). The short-form McGill Pain Questionnaire. Pain.

