# Supplementary material for: Ultrasound-Based Assessment of Shoulder Soft Tissue Alterations in Young Adults Performing Upper Limb Weight Training: A Cross-Sectional Study
Source: J Funct Morphol Kinesiol. 2026 Jan 1;11(1):23. doi: 10.3390/jfmk11010023 (PMC12821532; doi:10.3390/jfmk11010023)
Supplement: Supplementary file 1 [file jfmk-11-00023-s001.zip › Supplementary Table S1. Training Characteristics.pdf]

Supplementary Table S1. Training characteristics of weight-training participants (n = 15)

ID	Sex	Age (years)	Training experience (years) / Training Goal	Training type	Exposure (total / weekly sessions)	Average volume and intensity (sets × repetitions × exercises)	Adherence (%) / missed sessions	Main exercises performed
22	M	33	13 years / Increase muscle strength and hypertrophic adaptation	Strength/Hypertrophy	14 (3/week)	5 × 8 × 3	100 / 0	Bench press, overhead press, pull-ups
24	M	34	14 years / Strength gain and toning	Strength/Hypertrophy	12 (2/week)	4 × 10 × 3	87.5 / 1	Bench press, biceps curls, shoulder press
25	F	27	5 years / Maintain and improve overall fitness and muscle tone	Functional / General conditioning	12 (2/week)	3 × 12 × 3	100 / 0	Push-ups, resistance bands, cable rows
28	M	40	20 years / Increase maximal strength and muscle mass	Strength/Hypertrophy	18 (3/week)	5 × 6 × 3	100 / 0	Bench press, pull-ups, shoulder press
30	M	42	4 years / Enhance functional fitness and muscle tone	Functional / General conditioning	12 (2/week)	3 × 15 × 3	87.5 / 1	TRX rows, push-ups, kettlebell swings
31	M	45	10 years / Enhance overall strength and endurance performance	Strength/Hypertrophy	24 (4/week)	5 × 8 × 3	100 / 0	Bench press, overhead press, pull-downs
35	F	38	8 years / Maintain overall fitness and functional strength	Functional / General conditioning	12 (2/week)	3 × 12 × 3	75 / 2	Medicine ball throws, push-ups, rows
45	F	29	5 years / Increase muscular strength and tone	Strength/Hypertrophy	18 (3/week)	5 × 8 × 3	87.5 / 1	Bench press, biceps curls, military press
53	M	32	9 years / Develop maximal strength and muscle hypertrophy	Strength/Hypertrophy	30 (5–6/week)	5–8 × 8–20 × 4	100 / 0	Shoulder presses, lateral raises, free-weight work

57	M	44	7 years / Increase muscle strength and hypertrophic adaptation	Strength/Hypertrophy	30 (5/week)	4 × 10 × 4	100 / 0	Bench press, lat pulldown, rows, dumbbell shoulder work
62	M	37	11 years /Develop strength, power, and muscle hypertrophy	Strength/Hypertrophy	30 (5/week)	10 × 10 × 4	80/6	Snatch, squats, bench press, box jumps
66	M	28	5 years / Increase muscle strength and hypertrophy	Strength/Hypertrophy	30 (5/week)	4 × 10 × 4	100 / 0	Leg press, squats, barbell presses, rows
69	M	36	6 years / Develop maximal strength and muscle mass	Strength/Hypertrophy	24 (4/week)	5 × 6 × 3	100 / 0	Bench press, overhead press, deadlift
71	F	42	12 years /Maintain functional fitness and muscular endurance	Functional / General conditioning	12 (2/week)	3 × 15 × 3	87.5 / 1	Circuit training, push-ups, rows
72	M	36	11 years / Increase muscular strength and hypertrophy	Strength/Hypertrophy	18 (3/week)	4 × 8 × 3	100 / 0	Bench press, pull-ups, overhead press

Notes: All data correspond to the six-week monitoring period, during which each participant completed a minimum of eight upper-limb resistance sessions. Attendance (%) is calculated considering these eight scheduled sessions. Mean load (% 1RM) was estimated according to the predominant training objective. The data presented are modeled for illustrative purposes and align with the methodological description of the study.
