# Supplementary material for: Ultrasound-Based Assessment of Shoulder Soft Tissue Alterations in Young Adults Performing Upper Limb Weight Training: A Cross-Sectional Study
Source: J Funct Morphol Kinesiol. 2026 Jan 1;11(1):23. doi: 10.3390/jfmk11010023 (PMC12821532; doi:10.3390/jfmk11010023)
Supplement: Supplementary file 1 [file jfmk-11-00023-s001.zip › Supplementary Table S2. Non-Weight Training Physical Activity.pdf]

Supplementary Table S2. Physical activity profile of non-weight-training participants (n = 51)

ID	Type of Physical Activity	Sessions/month	Avg. Duration (min/session)	Intensity Level	Example Activities
1	None (sedentary)	0	0	—	—
2	Walking	3	30	Low	Commuting or leisure walk
3	Yoga	2	45	Low	Vinyasa-style class
4	Pilates	2	50	Low	Mat Pilates routine
5	Walking	4	25	Low	Short daily walks
6	Aerobics	2	40	Low	Low-impact gym class
7	Swimming	2	40	Low	Recreational swimming
8	None (sedentary)	0	0	—	—
9	Cycling	3	60	Low	Stationary or outdoor cycling
10	Walking	2	30	Low	Walking to work
11	Yoga	2	40	Low	Guided session at gym
12	Walking	3	25	Low	Evening walk
13	Swimming	2	45	Low	Recreational lap swimming
14	Pilates	2	45	Low	Studio Pilates session
15	Walking	2	30	Low	Leisure walk in park
16	Cycling	3	50	Low	Stationary bike training
17	Yoga	2	45	Low	Morning stretching and balance
18	Walking	3	25	Low	Walking to transport stop
19	None (sedentary)	0	0	—	—
20	Pilates	2	50	Low	Rehabilitation-oriented exercises
21	Walking	2	30	Low	Commuting on foot
22	Yoga	3	40	Low	Hatha yoga routine
23	Swimming	2	45	Low	Light training at pool
24	Cycling	2	45	Low	Weekend cycling route
25	Walking	3	30	Low	Leisure walking
26	None (sedentary)	0	0	—	—
27	Pilates	2	40	Low	Mat and reformer session
28	Walking	4	25	Low	Evening walk after work
29	Yoga	2	45	Low	Vinyasa flow
30	Cycling	3	60	Low	Outdoor recreational cycling
31	Walking	2	30	Low	Short commute
32	Pilates	2	45	Low	Rehabilitation focus
33	Walking	3	25	Low	Light exercise
34	Swimming	2	40	Low	Lap swimming
35	Yoga	2	45	Low	Restorative yoga
36	Walking	2	20	Low	Short daily walk
37	Cycling	3	60	Low	Recreational cycling
38	Walking	4	30	Low	Regular exercise walking
39	Yoga	2	40	Low	Gentle yoga flow
40	Pilates	2	50	Low	Core strengthening session
41	Walking	3	25	Low	Neighborhood walk
42	None (sedentary)	0	0	—	—
43	Cycling	3	60	Low	Weekend cycling activity
44	Walking	2	30	Low	Commuting to workplace
45	Yoga	2	45	Low	Evening relaxation class
46	Pilates	2	45	Low	Mat-based session
47	Walking	3	25	Low	Light aerobic walking
48	Swimming	2	40	Low	Low-impact pool exercise
49	Walking	3	30	Low	Leisure walking
50	Yoga	2	40	Low	Gentle yoga mobility
51	Walking	2	30	Low	Daily short walks

Notes: Data refer to participants classified as non-weight-training individuals. Activities were categorized according to self-reported frequency and duration during the six-week monitoring period. To ensure clear methodological separation from resistance exercise, all activities involving upper-limb movement (e.g., swimming, Pilates, yoga) were conservatively classified as low-intensity. Frequency was expressed as sessions per month to better reflect the low and irregular activity patterns of non-weight-training participants. Participants performing resistance exercises fewer than two times per week were excluded from the weight-training category.
